# Acquisition of anti-phosphatidylserine IgM and IgG antibodies by infants and their mothers over time in Uganda

**DOI:** 10.3389/fimmu.2024.1416669

**Published:** 2024-07-26

**Authors:** Muyideen Kolapo Tijani, Bandar Hassan Saleh, Allan Lugaajju, Lena Danielsson, Kristina E. M. Persson

**Affiliations:** ^1^ Division of Clinical Chemistry and Pharmacology, Department of Laboratory Medicine, Lund University, Lund, Sweden; ^2^ Department of Clinical Microbiology and Immunology, King Abdulaziz University, Jeddah, Saudi Arabia; ^3^ College of Health Sciences, Makerere University, Kampala, Uganda; ^4^ Clinical Chemistry and Pharmacology, Laboratory Medicine, Office for Medical Services, Region Skåne, Lund, Sweden

**Keywords:** phosphatidylserine, IgM, IgG, Plasmodium falciparum, malaria, infants

## Abstract

**Background:**

Production of anti-phosphatidylserine (anti-PS) antibodies has been associated with malaria and can aggravate pathology. How these autoantibodies develop during early childhood in a malaria context is not known. We examined levels of anti-PS IgG and IgM antibodies in a longitudinal cohort of mother-baby pairs during birth, in the infants at 2.5, 6 months, and in mothers and their babies at 9 months postpartum.

**Results:**

There was no difference between levels of anti-PS IgG in cord blood and the mothers’ peripheral blood at birth. However, anti-PS IgM levels were significantly higher in the mothers compared to the infants’ cord blood, and IgM levels were steadily increasing during the first 9 months of the infants’ life. In infants that had the highest anti-PS IgM levels at birth, there was a decline until 6 months with a rise at 9 months. Infants that possessed high anti-PS IgG at birth also exhibited a progressive decline in levels. When anti-PS were correlated to different fractions of B-cells, there were several correlations with *P. falciparum* specific atypical B cells both at birth and at 2.5 months for the infants, especially for anti-PS IgM. Anti-PS also correlated strongly to C1q-fixing antibodies at birth.

**Conclusion:**

These results show that anti-PS IgG acquired by mothers could be transferred transplacentally and that IgM antibodies targeting PS are acquired during the first year of life. These results have increased the knowledge about autoimmune responses associated with infections in early life and is critical for a comprehensive understanding of malaria vaccine functionality in endemic areas.

## 1 Introduction

Malaria still constitutes a huge public health challenge in affected areas with a global estimate of 249 million clinical cases and 608,000 deaths for the year 2022 (1). There was a 5 million increase in the number of cases when compared with 2021 and Ethiopia (+1.3 million), Nigeria (+1.3 million) and Uganda (+597,000) were the main contributing countries in Africa (1). Most of the deaths in malaria are caused by the parasite *Plasmodium falciparum.* Pregnant women and children undoubtedly carry an extra burden of malaria in endemic areas through maternal anemia, spontaneous abortion, preterm deliveries, lower birthweight and higher mortality and morbidity in young children (1–4). Apart from the effect of direct interactions of the parasite with the host that mediate some of these pathological consequences, anti-phospholipid autoantibodies associated with the infection may further complicate the situation (5–9). Phosphatidylserine (PS) is an inner leaflet membrane phospholipid and the exteriorization of PS, which is a marker of eryptosis, has been demonstrated on both infected and non-infected RBCs during *in vitro* cultures of *P. falciparum* (10, 11). Consequently, antibodies targeting PS, anti-PS, have been demonstrated in individuals naturally infected with different *Plasmodium* parasite species known to infect humans (6, 8, 12, 13). RBC surface remodeling where PS is temporarily exposed by immature gametocyte (14) may also enhance anti-PS antibody production during malaria.

The functions of anti-PS antibodies are not completely known, but malaria cohort studies from diverse endemic areas have established a relationship between anemia and anti-PS antibodies (6, 12, 13, 15, 16). Apart from anemia, anti-PS antibodies have also been linked with acute kidney damage and post-discharge mortality in children living in an endemic area in Kenya (8). Anti-PS antibodies have also been demonstrated in pregnant women living in endemic areas where multigravidae women that have not experienced placental malaria have the tendency to have lower levels of these antibodies compared to primigravidae and secundigravidae women (17, 18). Generally, anti-PS antibodies have been associated with pathological functions (16) and in pregnant women, anti-PS antibodies and other antiphospholipid antibodies commonly found in autoimmune diseases have been associated with various obstetric pathologies and serious adverse impacts on the fetus (7, 19).

The mechanism through which anti-PS antibodies develop during malaria and other infections is not clear, but exposure of PS in connection with malaria-associated hemolysis or appearance of new proteins on the infected erythrocyte surface is thought to be plausible pathways. Indeed, hemolysis triggered by *Plasmodium* or viral infections have been shown to be associated with the expansion of polyclonal B cells (20). Anti-PS antibodies have been demonstrated to be produced by atypical B cells (13, 21), which are commonly found in individuals living in malaria endemic areas (22, 23). A B cell population of similar description to the atypical B cells, called Double Negative, has also been connected to systemic lupus erythematosus (SLE) (24).

Autoantibodies are generally understudied in both infants and older children (25). There is lack of information about the development of infection-associated anti-PS antibodies during the early phase of life. In this paper we investigated the dynamics of IgM and IgG anti-PS antibodies in a longitudinal cohort of mother-baby pairs living in a malaria endemic area in Uganda.

## 2 Materials and methods

### 2.1 Study population

This study involved a longitudinal collection of samples from mother-infant pairs living in Kasangati, Uganda. Blood samples were collected from mothers during birth and after 9 months. Samples were collected from the newborns at birth (cord blood) and subsequently at 2,5, 6, and 9 months. The pregnant women received at least one dose of sulfadoxine-pyrimethamine as part of the intermittent preventive treatment in pregnancy (IPTp). The ethical approvals for this study were obtained from the Makerere University School of Medicine Research and Ethics Committee, The Uganda National Council of Science and Technology (2007–045), and Regionala Etikprövningsnämnden in Stockholm, Sweden (2011/132–31/3). All participants or their guardians signed informed consent forms before samples were collected.

### 2.2 Detection of anti-PS and complement-fixing antibodies using Enzyme linked immunosorbent assay

IgM and IgG antibodies produced against PS in plasma samples were measured on separate plates using an ELISA kit (Orgentec, Germany) according to the manufacturer’s instructions. Samples (diluted 1:100), controls and calibrators were added to plates that had been precoated with PS and then incubated for 30 minutes at room temperature (RT). After washing the plates twice, HRP-labelled anti-human IgG or IgM was added to the plates followed by incubation at RT for 15 min. Plates were washed again and color was developed by adding TMB (3,3’,5,5’-tetramethylbenzidine) and then incubated for 15 min at RT. Stop solution was added to stop the reaction and absorbance read at 450 nm. The concentration of anti-PS was determined from a standard curve created by the manufacturer. Complete sets of the same mother-baby longitudinal samples were run on the same plate to reduce inter-assay variations.

Levels of C1q-fixing antibodies targeting merozoite crude antigens were determined as recently reported elsewhere (26). Briefly, Maxisorp 96-well plates (Nunc, Roskilde, Denmark) were coated with homogenized merozoite extract in phosphate-buffered saline (PBS), washed with washing buffer (PBS-0.01% Tween 20), then blocked with 1% casein (ThermoFisher Scientific, Rockford, IL, USA) in PBS. Controls and test plasma samples diluted 1:50 in 0.1% casein/washing buffer were added to the wells in duplicates after washing and then incubated overnight. Plates were washed again and Human C1q (10 μg/mL) (EMD Millipore Corp, USA) was added followed by incubation and washing. This was followed by addition of rabbit anti-C1q IgG antibodies (Beeson lab) 1:2000 and anti-rabbit IgG (H+L)-horseradish peroxidase 1:3000 (BioRad Laboratories, USA), with in-between and final washing. Color was then developed by adding TMB substrate (TMB One Solution ready-to-use, Promega, Madison, WI, USA). Color development was stopped with 1 M H_2_SO_4_ at 30 minutes and absorbance read at 450 nm (Multiskan Sky, ThermoFisher Scientific, Rockford, IL, USA). Longitudinal samples from the same mother-baby pairs were always analyzed on the same plates to limit inter-assay variations.

### 2.3 *P. falciparum*-specific B cells immunophenotyping by flow cytometry

*P. falciparum*-specific B cells were determined by their binding to conjugates of carboxyl Quantum dots and trophozoite/schizont parasite ghost cells (GiRBC-Qdot conjugate) (27, 28). *P. falciparum-*specific B cells were immunophenotyped using a flow cytometry protocol that has been described elsewhere (22). Briefly, approximately 1 × 10^6^ cryopreserved peripheral blood mononuclear cells, PBMCs, were thawed into RMPI 1640 containing 10% fetal bovine serum (FBS) and then sendimented by centrifuging at 330 g for 10 minutes. Sendimented cells were then washed in cold flow buffer (PBS/0.5% BSA/2 mM EDTA) and then resuspended in 100 µL flow buffer. Non-specific binding and background fluorescence were reduced by incubating PBMC samples with 1 µg Fc block (CD16/CD32mAb, Biolegend) for 5 minutes on ice. GiRBC-Qdot conjugate was then added, and incubated on ice for 30 min. The cells were washed and stained with CD19, CD20, CD27, FcRL4 and IgG antibodies for 30 min on ice. Cells were resuspended in 300 µL flow buffer after wasing and thereafter analysed on a LSRII flow cytometer (Becton–Dickinson Immunocytometry Systems, San Jose, USA). Acquired data was further processed using FLOWJO software (Tree Star Inc., San Carlos, CA, USA). B cell populations were defined as follows: naïve B cells (CD19+CD20+CD27−FcRL4 ± IgG−), plasma cells (CD19+CD20−CD27+FcRL4 ± IgG−), IgG memory B cells (CD19+CD20+CD27+FcRL4 ± IgG+), non-IgG+ memory B cells (CD19+CD20+CD27+FcRL4 ± IgG−), and atypical memory B cells (CD19+CD20+CD27-FcRL4 ± IgG+).

### 2.4 Statistical analyses

Mann-Whitney test and Wilcoxon matched-pairs signed rank were used to determine differences in antibody levels between unpaired and paired groups of samples, respectively. A linear mixed model was used to analyze the antibody responses in the infants over time. For each antibody response, two models were estimated, one to model how the antibody levels changed over time for infants and the other where the infants’ antibody levels were adjusted for their mothers’ antibody levels at birth. Additionally, linear mixed models were employed to investigate if antibody levels changed differently over time depending on whether the infants’ antibody level was high or low at birth. A high antibody response at birth was defined as a value in top 25% of values for each antibody response. Correlations were estimated using Pearson correlations for each time point and separately for mothers and infants. False discovery rate was used to adjust for multiple comparisons. Analyses were performed using R Statistical Software 4.1.2 (Foundation for Statistical Computing, Vienna, Austria) and GraphPad Prism version 10.0 (GraphPad Software, San Diego, California USA).

## 3 Results

### 3.1 Characteristics of study participants

A total of 131 mother-baby pairs were recruited into the study, but only 109 were followed from birth to 9 months. Due to insufficiency of plasma volumes, the number of samples involved in this study varied: mothers at birth (n =34), infants at birth (n = 34), infants at 2.5 months (n = 25), infants at 6 months (n = 24), infants at 9 months (n = 26), mothers at 9 months (n = 30). All the participants were healthy with no observable symptoms for any diseases and the mothers did not have any obstetric complications during birth. The mean age and gravidity of the mother involved in the current study were 24.1 and 2.6, respectively.

### 3.2 Anti-PS IgG and IgM antibody levels in infants over time

**Figure 1:**
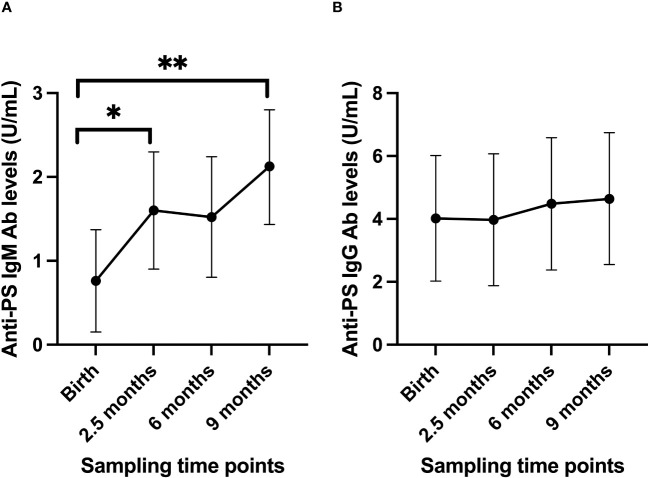
Longitudinal anti-PS IgM **(A)** and IgG **(B)** antibody profiles in infants from birth to 9 months postpartum. Using the values at birth as references, there were significant increases in anti-PS IgM antibody levels (p-values 0.036 at 2.5 months and 0.001 at 9 months), while for IgG there were no significant changes (p values were 0.97 at 2.5 months, 0.74 at 6 months and 0.62 at 9 months). Dots represent estimated mean and whiskers represent 95% confidence intervals. '*' is < 0.05 and '**' is < 0.01.

The estimated mean of anti-PS IgM levels increased steadily from birth to 9 months (**Figure 1A**). On the contrary, levels of anti-PS IgG antibody did not change significantly from birth through to 9 months of age of the infants (**Figure 1B**). To account for any effects that the levels of anti-PS IgM and IgG antibodies in the mothers might have on the levels of the antibodies in the infants, the levels of anti-PS antibodies in the infants were adjusted for their mothers’ anti-PS antibody levels using a linear mixed model. The adjustment did not reveal any differences in the longitudinal patterns already established for anti-PS IgM or IgG antibodies in the infants.

**Figure 2:**
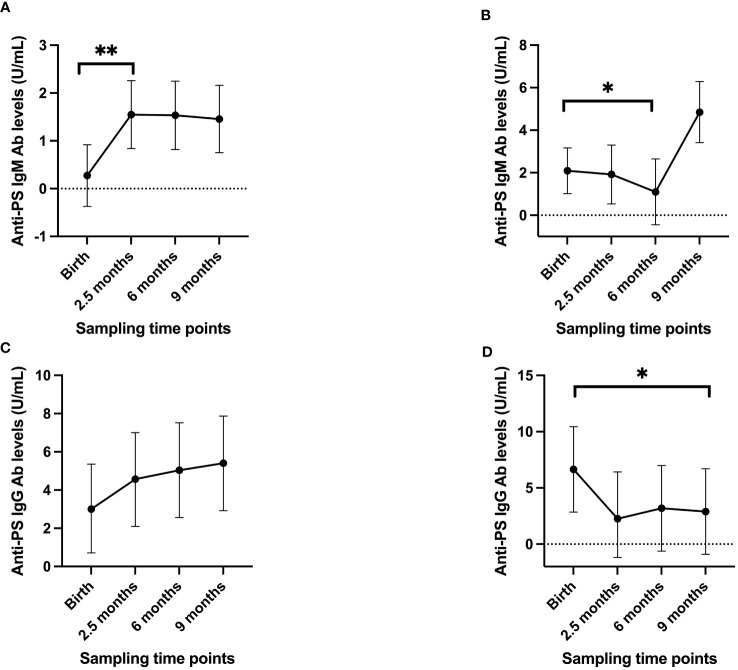
Longitudinal antibody profiles of infants when divided into high/low responders, with the high IgM/IgG responders having antibody levels in the top 25% of values at birth while the remaining were considered to be low responders. **(A)** low IgM responders **(B)** high IgM responders **(C)** low IgG responders **(D)** high IgG responders. Dots represent estimated mean and whiskers represent 95% confidence intervals. '*' is < 0.05 and '**' is < 0.01.

To gain further insights into the nature of the development of anti-PS IgM and IgG antibodies, the infants were further grouped into high and low responders based on their anti-PS antibody levels at birth. Those in the top 25% of the anti-PS antibody levels were categorized as high responders and the rest as low responders. The linear mixed model showed that low responders for anti-PS IgM antibodies had a drastic increase in antibody level at 2.5 months (p = 0.001), while the level after that remained almost constant at 6 months and 9 months, but significantly higher than the levels recorded at birth (**Figure 2A**). High anti-PS IgM responders exhibited a gradual decay in anti-PS IgM antibody levels, which became significant at 6 months of age (p = 0.02), but there was an insignificant increase in level at 9 months (p = 0.106) (**Figure 2B**). For IgG, the low responders showed a slight increase in anti-PS IgG from birth that was not statistically significant (**Figure 2C**). High anti-PS IgG responders showed a decrease in antibody levels overtime that became significant when the infants reached 9 months of age (p = 0.04) (**Figure 2D**). Adjustment for maternal levels of anti-PS IgM and IgG antibodies at birth did not alter the observed patterns in either the low or high responders.

### 3.3 Comparisons of infants and mothers’ anti-PS antibodies at birth and 9 months post-partum

**Figure 3:**
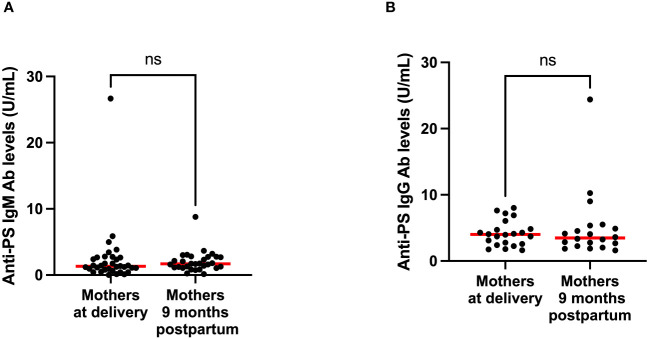
Comparison of median anti-PS IgM **(A)** and IgG **(B)** antibodies for mothers at delivery and 9 months postpartum. Red lines represent the median and ‘ns’ not statistically significant (Wilcoxon matched pairs signed rank).

**Figure 4:**
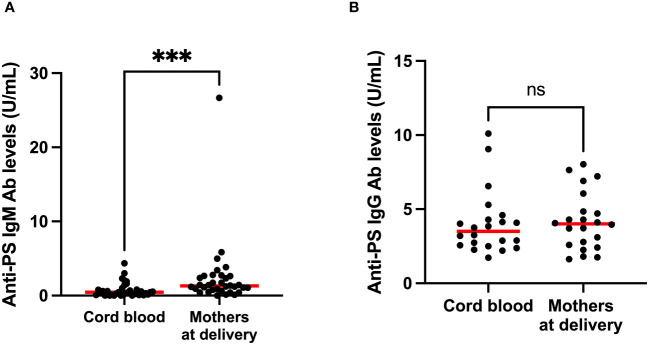
Comparison of median anti-PS IgM **(A)** and IgG **(B)** antibodies in cord blood and mothers at delivery. Red lines represent the median, ‘ns’ not statistically significant and *** represents statistical significance at p < 0.001 (Mann-Whitney).

**Figure 5:**
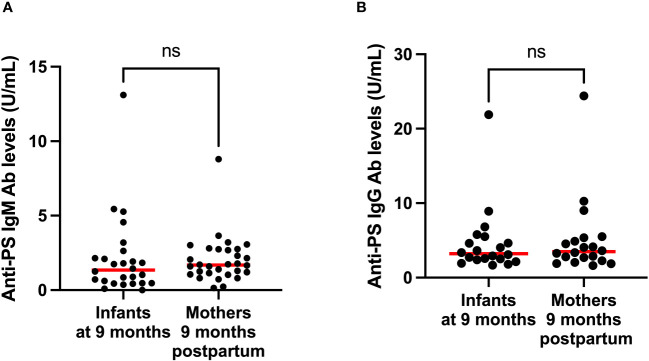
Comparison of median anti-PS IgM **(A)** and IgG **(B)** antibodies of infants and mothers 9 months postpartum. Red lines represent the median and ‘ns’ not statistically significant (Mann-Whitney).

To gain insight into the contributions of childbirth associated factors to the generation of anti-PS IgM and IgG antibodies and possible transplacental transfer of antibodies, maternal levels of these antibodies were compared with levels in cord blood between birth and 9 months post-partum. There was no significant difference between levels of anti-PS IgM and IgG at birth and 9 months post-partum in mothers (**Figures 3A, B**). Unsurprisingly, IgM was significantly lower in infants (cord) than in mothers at birth (p = 0.0003) (**Figure 4A**). Meanwhile, there was no difference between the cord blood and mothers anti-PS IgG antibody levels at birth (**Figure 4B**). At 9 months, the infants had acquired anti-PS IgM and IgG antibody levels comparable to their mothers at 9 months postpartum (**Figures 5A, B**).

### 3.4 Association between anti-PS and complement-fixing antibodies

Since IgG antibodies that fix C1q had earlier been shown to be transplacentally transferred in the same mother-baby pairs (26) involved in this current study, correlation analysis was carried out to determine the relationship between functional malaria-specific, C1q-fixing antibodies and anti-PS antibodies. There was a strong correlation (ρ = 0.757, p < 0.001) between C1q-fixing and anti-PS IgG antibodies in the infants at birth. No significant correlation was found at any other time points in the mothers or infants for IgG or IgM antibodies (**Table 1**).

**Table 1:** Correlation between anti-PS and C1q-fixing antibodies.

	Mothers at birth	Infants at birth	Infants at 2.5 months	Infants at 6 months	Infants at 9 months	Mothers at 9 months
C1q-fixing IgG	ρ (Adjusted p value)	ρ (Adjusted p value)	ρ (Adjusted p value)	ρ (Adjusted p value)	ρ (Adjusted p value)	
Anti-PS IgM	0.140 (0.705)	-0.001 (0.996)	-0.119 (0.777)	-0.289 (0.349)	0.146 (0.688)	0.203 (0.524)
Anti-PS IgG	0.309 (0.376)	**0.757 (<0.001)**	0.009 (0.978)	-0.132 (0.777)	0.251 (0.488)	0.467 (0.116)

Bold indicates significant correlation coefficients and p values.

### 3.5 Association between B cell subtypes and anti-PS IgM and IgG antibody levels

To further explore the relationship between anti-malaria immune response development and autoimmune antibody response, we calculated the correlation between B cells subtypes that had earlier been reported (22) and anti-PS IgM and IgG antibody levels (**Table 2**). At birth anti-PS IgM correlated with total IgG+ memory B cells (MBC) (p = 0.005) total atypical B cells (< 0.001), Pf+ IgG+ MBC (p = 0.04), Pf+ atypical MBC (<0.001) and Pf+ naïve B cells (p = 0.04). At 2.5 months anti-PS IgM only correlated with total atypical B cells (p < 0.001) and Pf+ atypical B cells (p = 0.001). There was no correlation between anti-PS IgM and all the B cell subtypes at 6 months while there was correlation with non-IgG MBC (p = 0.043) and total Pf+ B cells (p = 0.023) at 9 months. There was no correlation between anti-PS IgG antibodies and B cells at birth and 6 months, but there was correlation with total atypical B cells (p = 0.001), Pf+ atypical B cells (p < 0.001) at 2.5 months and Pf+ plasma cells (p = 0.002) at 9 months.

**Table 2:** Correlation between B cells types and anti-PS IgM and IgG antibodies.

	Mothers at birth	Infants At birth	Infants at 2.5 months	Infants at 6 months	Infants at 9 months	Mothers at 9 months
Anti-PS IgM	ρ (Adjusted p value)	ρ (Adjusted p value)	ρ (Adjusted p value)	ρ (Adjusted p value)	ρ (Adjusted p value)	ρ (Adjusted p value)
IgG MBC Non-IgG MBC Atypical MBC Naïve B Cells Plasma cells Total B cells Pf+ IgG MBC Pf+ non-IgG MBC Pf+ Atypical MBC Pf+ Naïve B cells Pf+ Plasma cells Total Pf+ B cells	**-**0.03 (0.947) -0.056 (0.894) -0.106 (0.808) 0.132 (0.773) -0.014 (0.975) -0.146 (0.723) -0.106 (0.808) -0.128 (0.777) -0.131 (0.776) 0.281 (0.323) 0.013 (0.975) -0.024 (0.952)	**0.588 (0.005)** 0.174 (0.592) **0.803 (<0.001**) -0.286 (0.300) 0.102 (0.803) -0.221 (0.446) **0.455 (0.044)** 0.320 (0.206) **0.821 (<0.001**) **-0.463 (0.040)** -0.027 (0.952) -0.190 (0.540)	0.512 (0.064) 0.378 (0.229) **0.768 (<0.001**) -0.493 (0.079) 0.291 (0.404) -0.198 (0.623) 0.376 (0.233) 0.255 (0.438) **0.695 (0.001)** -0.416 (0.169) 0.185 (0.658) 0.519 (0.059)	0.403 (0.133) 0.217 (0.5209 0.276 (0.376) -0.354 (0.206) 0.136 (0.724) -0.067 (0.875) 0.475 (0.059) 0.214 (0.527) 0.207 (0.549) -0.450 (0.079) 0.108 (0.805) 0.446 (0.083)	0.344 (0.198) **0.482 (0.043**) 0.154 (0.668) -0.409 (0.104) 0.079 (0.838) 0.454 (0.061) 0.359 (0.174) 0.188 (0.576) 0.139 (0.705) -0.434 (0.078) -0.017 (0.973) **0.521 (0.023)**	-0.212 (0.524) -0.117 (0.808) -0.125 (0.784) 0.247 (0.452) -0.051 (0.909) -0.026 (0.952) -0.200 (0.559) -0.155 (0.705) -0.122 (0.793) 0.325 (0.240) -0.103 (0.817) 0.343 (0.207)
Anti-PS IgG						
IgG MBC Non-IgG MBC Atypical MBC Naïve B Cells Plasma cells Total B cells Pf+ IgG MBC Pf+ non-IgG MBC Pf+ Atypical MBC Pf+ Naïve B cells Pf+ Plasma cells Total Pf+ B cells	-0.054 (0.915) -0.111 (0.827) 0.263 (0.499) -0.054 (0.915) -0.264 (0.499) 0.199 (0.668) -0.131 (0.808) -0.061 (0.908) 0.070 (0.890) 0.155 (0.777) -0.277 (0.468) -0.187 (0.700)	-0.185 (0.648) -0.045 (0.926) -0.252 (0.471) 0.179 (0.658) -0.093 (0.838) 0.277 (0.416) -0.165 (0.687) -0.064 (0.888) -0.201 (0.597) 0.176 (0.666) -0.113 (0.805) -0.055 (0.903)	0.132 (0.805) -0.072 (0.888) **0.795 (0.001**) -0.97 (0.843) 0.172 (0.714) 0.066 (0.900) 0.117 (0.824) -0.109 (0.819) **0.812 (<0.001)** -0.034 (0.954) 0.162 (0.732) 0.176 (0.706)	0.092 (0.838) 0.061 (0.900) 0.025 (0.966) -0.084 (0.858) 0.050 (0.920) 0.226 (0.554) 0.079 (0.867) -0.096 (0.837) 0.031 (0.954) -0.016 (0.973) 0.022 (0.968) 0.267 (0.450)	-0.126 (0.796) 0.151 (0.723) -0.105 (0.826) 0.044 (0.931) 0.400 (0.191) -0.117 (0.805) -0.163 (0.702) 0.166 (0.694) 0.026 (0.964) -0.022 (0.968) **0.707 (0.002)** 0.182 (0.663)	0.125 (0.817) 0.222 (0.616) 0.253 (0.524) 0.029 (0.952) -0.214 (0.639) -0.187 (0.705) 0.313 (0.400) 0.429 (0.173) 0.103 (0.852) -0.105 (0.852) -0.228 (0.602) 0.116 (0.825)

Bold indicates significant correlation coefficients and p values.

For the mothers, there were no significant correlations between anti-PS antibodies and B cells.

### 3.6 Comparison of anti-PS IgG/IgM according to gravidity

We measured the mothers´ anti-PS and analyzed the data according to primigravidae, secundigravidae and multigravidae (**Supplementary Figure S1**). We could not detect any significant differences depending on gravidity.

## 4 Discussion

The development of effective vaccines for malaria requires a comprehensive understanding of associated immune responses. Anti-PS antibodies contribute to malaria pathology by binding to uninfected RBCs causing anemia (6, 15). We demonstrate the acquisition of anti-PS IgM and IgG antibodies in healthy mother-baby pairs involved in a longitudinal study conducted in a malaria endemic area in Uganda.

Infants growing up in malaria endemic areas are exposed to malaria infections (29–31), but are protected for the first few months of life by transplacentally transferred antibodies targeting different malaria parasite antigens (32, 33). The characteristics of transferred antibodies are dependent on the prenatal exposure of the mothers (33) and it was recently demonstrated that complement-fixing IgG antibodies could be transferred from mothers to neonates (26). Our current results show that anti-PS IgG antibodies that could be associated with malaria infections were transplacentally transferred, possibly alongside other maternal antibodies as indicated by their correlation with merozoite specific complement fixing antibodies, to the neonates as there was no difference between levels of anti-PS IgG in the cord blood at birth and peripheral blood of the mothers. IgG transfer across the placenta is mediated by neonatal Fc receptors (FcRn) where the output may be affected by placental health, IgG subclass, mother’s total IgG, specific antibody levels, gestational age and antigen characteristics (34, 35). The cohorts in our study were generally healthy, without any obstetric complications, so we assume that these autoantibodies have been naturally transferred from the mothers. Anti-PS antibodies have been found to be lowest in multigravidae women compared to primigravidae and secundigravidae (17, 18), so it possible that gravidity could affect the levels of maternally transferred anti-PS antibodies. When we tried to analyze our data for gravidity (**Supplementary Figure S1**) we could not detect any significant differences depending on gravidity, but it might be due to too low number of samples. However, it is important to note that placental health could override our assertion here as multigravidae women with placental malaria could have comparatively higher levels of anti-PS antibodies (18). Neonates are also capable of some *in utero* antibody production when intact parasites (36) or parasite proteins (37, 38) cross the placenta and end up in their system. Such cases are rare, and antibodies produced because of such bridges of the mother-fetus barrier is negligible (39). Generally, IgG antibodies specific to different pathogens in the peripheral blood of the mothers correlate with that of the cord blood at birth (40, 41), but some of the antibodies may have been transferred according to the gestational age (42). Furthermore, anti-phospholipid antibodies in mothers that have anti-phospholipid syndrome and other autoantibodies are also transplacentally transferred to neonates (43–45). As expected, there was no evidence of transplacental transfer of anti-PS IgM antibodies as the levels in the cord blood were significantly lower compared to the mothers. When IgM is detected immediately after birth, they are usually linked to *in utero* production, even when there is no clear detection of the responsible pathogen. In a particular case, IgM specific to SARS-Cov-19 was detected only a few hours after birth, but all the newborns tested negative for the virus (46).

Our results show that the end of pregnancy/childbirth process have no impact on anti-PS IgM and IgG antibodies because levels of these antibodies did not change from birth to 9 months postpartum in the mothers. We could not find any previous studies that have considered the effect of childbirth on pathogen-specific, or pathogen associated autoantibodies, probably because there are no apparent connections. It is possible that we could have seen changes in antibody levels if we had checked sooner after childbirth as 9 months postpartum is a considerable long time in terms of antibody dynamics.

Our observation that anti-PS IgM antibodies generally increased over time is consistent with the notion of the babies´ own development of IgM antibodies as the babies encounter malaria since IgM is not transferable over the placenta. This agrees with some studies that showed that IgM antibodies specific to some malaria antigens increase in the early months of life in endemic areas (47–49). Since IgG is expected to have waned significantly at 6 to 9 months after birth (47, 48, 50), consistent levels of anti-PS IgG antibodies that we observed from birth to 9 months may indicate that infants started producing anti-PS IgG early enough so that the newly produced antibodies matched up with the maternally transferred antibodies. The pattern of anti-PS antibody development became clearer when the infants were split into low and high responders. High anti-PS IgM antibody responders experienced an antibody decline for some time before a later leap in level (that was not statistically significant), and low responders exhibited an initial leap in antibody and then stagnation. The factors controlling the changes in the IgM levels are not clear from our data, but we speculate that they are related to malaria infection and possibly other infections that elicit anti-PS antibodies, most importantly because all the children were assumedly born free of any autoimmune diseases. We further show that anti-PS IgG high responders declined overtime which is consistent with waning of maternal antibodies targeting malaria antigens (32, 47, 51) and this is in general true of maternal IgG antibodies with different antigen specificities (43). A follow-up of infants with maternally transferred autoantibodies also showed that they waned with time such that they were undetectable in some infants at 3 months (44). In contrary to high IgG responders, low responders exhibited the tendency to acquire more antibodies with time, which might be linked to their experience of infections.

Interestingly, in **Figure 5B**, there is one infant and one mother with exceptionally high values compared to the rest, aBd these are the corresponding mother-baby pair. Perhaps these individuals had both very recently been exposed to malaria because anti-PS antibody production could be triggered by the contact between parasite DNA and PS-antibody producing B cells, CD11c^+^T-bet^+^ B cells (21), although some studies have not found any correlation between parasitemia and anti-PS antibodies (12, 17). The mother was also a primigravida and primigravidae are more likely to have higher levels of anti-PS (17, 18), especially if placental malaria is not involved (18). However, there was no significant differences in anti-PS levels among women of different gravidity in this current study (**Supplementary Figure S1**). At earlier time points these individuals did not reveal particularly elevated levels, so we do not believe that it was due to a genetic condition. For the other figures there was no such connections.

Overall, the longitudinal antibody profiles of the infants suggest some kind of homeostatic mechanism that regulate anti-PS antibodies such that they are upregulated when low or down regulated when high. Furthermore, at 9 months postpartum, the infants have already acquired both IgM and IgG anti-PS antibodies comparable to their mothers’. For most antigen specificities, maternal antibodies are expected to be higher considering their immunological experience, but again maybe there is a mechanism to regulate or limit the levels of anti-PS antibodies associated with infections. This limited antibody production might be connected to the B cell sources of anti-PS antibodies, which has been shown to be atypical B cells (13, 21). Atypical B cells are dysfunctional B cell populations that expand during malaria (13, 15, 22), HIV (52), covid-19 (53, 54), and other chronic infections (23). The idea that atypical B cells are dysfunctional in all malaria cases remains controversial (55), as atypical B cells have been demonstrated to produce functional anti-malaria antibodies (56–58). We also confirmed the correlation between atypical B cells (both *P. falciparum* specific and total atypical) and anti-PS antibodies like in an earlier study (13), especially with IgM antibodies. This also reinforces our suggestion that acquisition of IgM antibodies could be related to early malaria infections encountered by these infants. The lack of consistent correlation between anti-PS antibodies and atypical B cells over time maybe due to the divergent roles of atypical B cells in producing auto anti-PS antibodies and functional anti-malaria antibodies such that the level of correlation observed at any time point is a measure of the preponderance of anti-PS producing atypical B cells. Our data also show some correlations of anti-PS with plasma cells. This is consistent with an earlier observation that major compartments of B cells (memory, naïve etc.), other than atypical B cells, also have cell populations with loss of CD21, high CD11c and T-bet (24), which are the main characteristics of cells that produce anti PS antibodies (21). This requires further studies where B cells from major compartments will be tested for anti-PS production *in vitro*.

In conclusion, we have provided evidence that indicates that anti-PS IgG autoantibodies are placentally transferred to neonates in individuals living in a malaria endemic area. We also demonstrated the dynamics of these autoantibodies during the early part of life of infants and their relationship with different B cell populations. We recommend a repeat of this study in bigger cohorts in different malaria endemic areas where the functional activities of anti-PS antibodies can also be clearly demonstrated.

## Data availability statement

The original contributions presented in the study are included in the article/**Supplementary Material**. Further inquiries can be directed to the corresponding author.

## Ethics statement

The studies involving humans were approved by Makerere University School of Medicine Research and Ethics Committee, The Uganda National Council of Science and Technology, Regionala Etikprövningsnämnden in Stockholm, Sweden. The studies were conducted in accordance with the local legislation and institutional requirements. Written informed consent for participation in this study was provided by the participants’ legal guardians/next of kin.

## Author contributions

MT: Conceptualization, Data curation, Formal analysis, Investigation, Methodology, Supervision, Writing – original draft, Writing – review & editing. BS: Formal analysis, Investigation, Methodology, Writing – review & editing, Data curation. AL: Data curation, Investigation, Writing – review & editing. LD: Investigation, Writing – review & editing, Conceptualization, Methodology. KP: Conceptualization, Investigation, Methodology, Writing – review & editing, Formal analysis, Funding acquisition, Project administration, Resources, Supervision, Validation, Visualization.

## Supplementary material

Supplementary Figure 1 Comparison of median anti-PS IgM at birth **(A)** and 9 months postpartum **(B)**; and median anti-PS IgG at birth **(C)** and 9 months postpartum between women of different gravidity. Red lines represent the median and ‘ns’ not statistically significant (Kruskal-Wallis).
