# Supplementary figures and images for: Acquisition of anti-phosphatidylserine IgM and IgG antibodies by infants and their mothers over time in Uganda
Source: Front Immunol. 2024 Jul 26;15:1416669. doi: 10.3389/fimmu.2024.1416669 (PMC11310174; doi:10.3389/fimmu.2024.1416669)

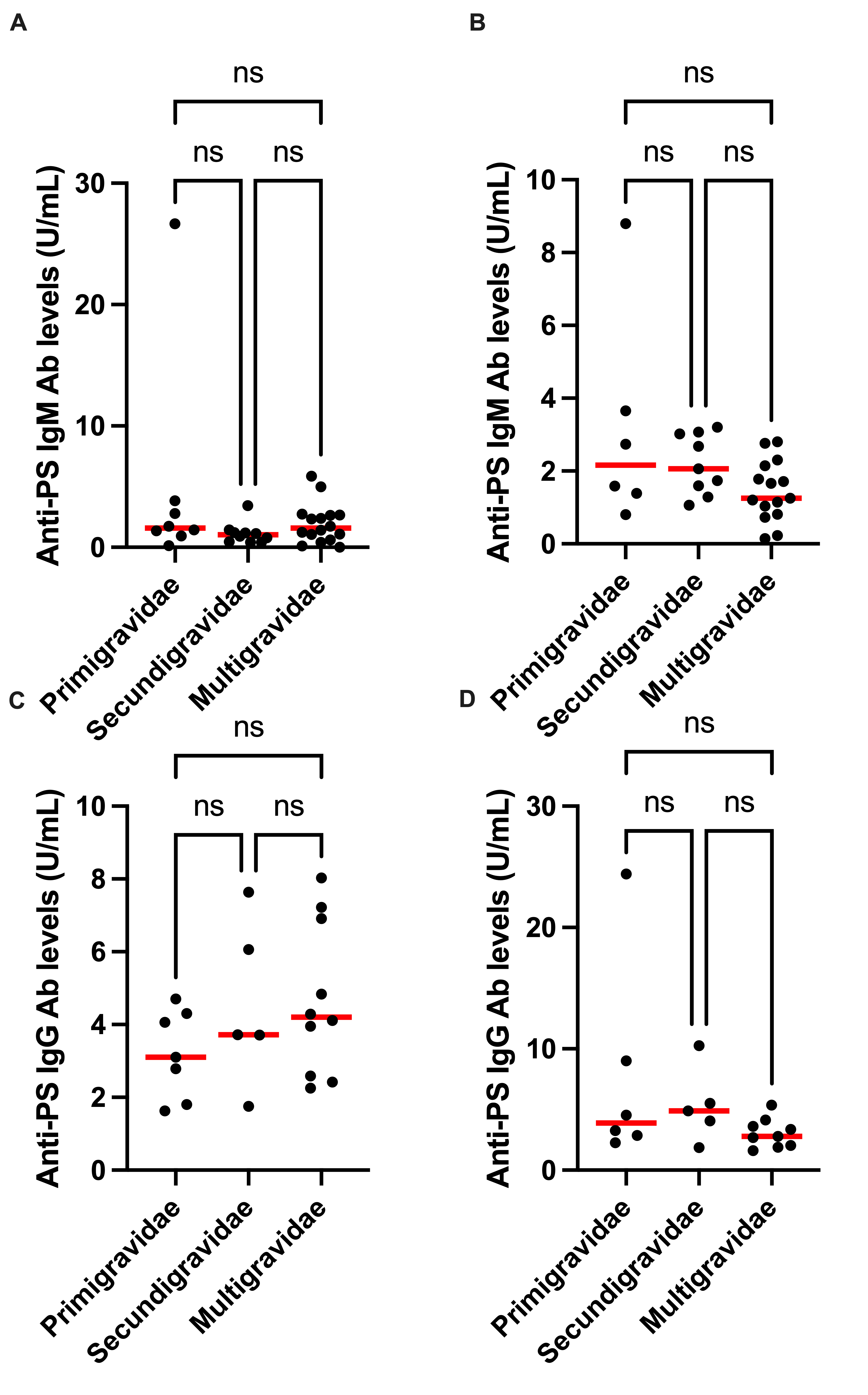

Supplement: Supplementary Figure 1 — Comparison of median anti-PS IgM at birth (A) and 9 months postpartum (B); and median anti-PS IgG at birth (C) and 9 months postpartum between women of different gravidity. Red lines represent the median and ‘ns’ not statistically significant (Kruskal-Wallis). [file Image_1.tiff]
